# Ultrastructural and Immunohistochemical Detection of Hydroxyapatite Nucleating Role by rRNA and Nuclear Chromatin Derivatives in Aortic Valve Calcification: In Vitro and In Vivo Pro-Calcific Animal Models and Actual Calcific Disease in Humans

**DOI:** 10.3390/ijms24032667

**Published:** 2023-01-31

**Authors:** Antonella Bonetti, Magali Contin, Maurizio Marchini, Fulvia Ortolani

**Affiliations:** Department of Medicine, University of Udine, I-33100 Udine, Italy

**Keywords:** aortic valve calcification, aortic valve interstitial cells, ectopic calcification, rRNA, ribosomes, nuclear chromatin, ultrastructure

## Abstract

Calcification starts with hydroxyapatite (HA) crystallization on cell membranous components, as with aortic valve interstitial cells (AVICs), wherein a cell-membrane-derived substance containing acidic phospholipids (PPM/PPLs) acts as major crystal nucleator. Since nucleic acid removal is recommended to prevent calcification in valve biosubstitutes derived from decellularized valve scaffolds, the involvement of ribosomal RNA (rRNA) and nuclear chromatin (NC) was here explored in three distinct contexts: (i) bovine AVIC pro-calcific cultures; (ii) porcine aortic valve leaflets that had undergone accelerated calcification after xenogeneic subdermal implantation; and (iii) human aortic valve leaflets affected by calcific stenosis. Ultrastructurally, shared AVIC degenerative patterns included (i) the melting of ribosomes with PPM/PPLs, and the same for apparently well-featured NC; (ii) selective precipitation of silver particles on all three components after adapted von Kossa reactions; and (iii) labelling by anti-rRNA immunogold particles. Shared features were also provided by parallel light microscopy. In conclusion, the present results indicate that rRNA and NC contribute to AVIC mineralization in vitro and in vivo, with their anionic charges enhancing the HA nucleation capacity exerted by PPM/PPL substrates, supporting the concept that nucleic acid removal is needed for valve pre-implantation treatments, besides better elucidating the modality of pro-calcific cell death.

## 1. Introduction

Since the 1970s, two lines of thought on valve calcification have been followed, one ascribing the pro-calcific process to alterations of the extracellular matrix components [1,2,3,4,5,6,7,8] and the other supporting the concept of cell-primed mineralization, with subsequent involvement of the extracellular matrix associated with the release of cell-derived “matrix vesicles” [9,10,11,12,13,14,15,16,17]. Namely, “calcium-phospholipid-phosphate complexes” made of cell-membrane-derived acidic phospholipids were identified as major hydroxyapatite (HA) crystal nucleators at the level of both mineralizing valve cells and their vesicular byproducts [11,14,15], according to calcification processes analogous to those occurring in hard tissues. This view of cell-derived lipids as major determinants in valve mineralization is actually supported by the fact that (i) lipid oxidative alterations are associated with calcification enhancement [18,19] and (ii) calcification prevention is achieved by lipid extraction [20,21,22,23] or complete cell removal [24,25,26,27,28] from valve tissues. Accordingly, in our previous investigations on porcine aortic valve leaflets (pAVLs) subjected to calcific induction in vivo [29,30,31,32,33,34,35] and bovine aortic valve interstitial cells (bAVICs) cultured under pro-calcific conditions [33,34,36,37,38], mineralization was found to depend on a peculiar degenerative process characterized by overall cell membrane lysis. Briefly, organelle disappearance was paralleled by the intracellular accumulation of an acidic-phospholipid-containing, phthalocyanine-positive material (PPM), the progressive spreading of which toward the edges of mineralizing cells and their vesicular debris gave rise to phthalocyanine-positive layers (PPLs) acting as major HA nucleators, as revealed by original von Kossa silver staining adapted to electron microscopy. In the extracellular matrix, both collagen fibrils and elastin fibers were found to mineralize only once engulfed by PPL-derived material. An analogous degenerative process was found for human aortic valve leaflets (hAVLs) affected by calcific stenosis [33,39]. Although nucleic acids are another relevant anionic cellular component in addition to negatively charged phospholipids, their possible involvement in AVIC calcification has thus far been neglected or merely mentioned in passing. The only study expressly reporting valve calcification onset at the nuclear level is that by Girardot and colleagues [40], in which traditional von Kossa silver staining in light microscopy was used to explore the effects of glutaraldehyde fixation on valve leaflets subjected to xenogeneic subdermal implantation. However, this optical approach cannot allow a reliable identification of the cell areas which are effectively masked by silver precipitates. Using traditional von Kossa silver reactions, two other studies have reported the occurrence of nuclear calcification in tissues other than cardiovascular ones, involving necrotic cells of the rat kidney parenchyma after ischemia induction [41] and, recently, brain cells of patients affected by Alzheimer’s disease undergoing abnormal accumulation of hyperphosphorylated tau protein [42]. In the present study, ultrastructural and immunohistochemical analyses revealed that degenerating ribosomes and the inherent ribosomal RNA (rRNA) contribute to forming PPM/PPL substrates, concurrently enhancing their capacity for HA nucleation in (i) primary cultures of bAVICs after pro-calcific stimulation, (ii) pAVLs that have undergone accelerated calcification after implantation into rat *subcutis*, and (iii) hAVLs affected by calcific stenosis. Moreover, the histochemical visualization of calcium binding sites revealed that nuclear chromatin (NC) is an additional HA nucleation site in all three calcific conditions.

## 2. Results

**Figure 1:**
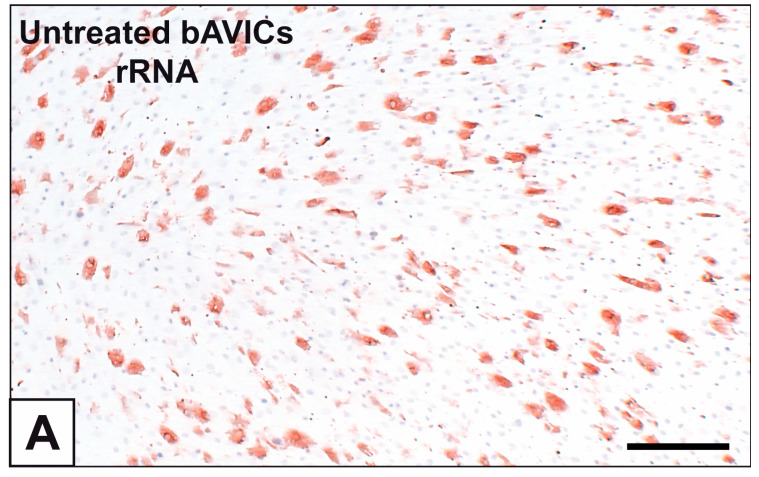

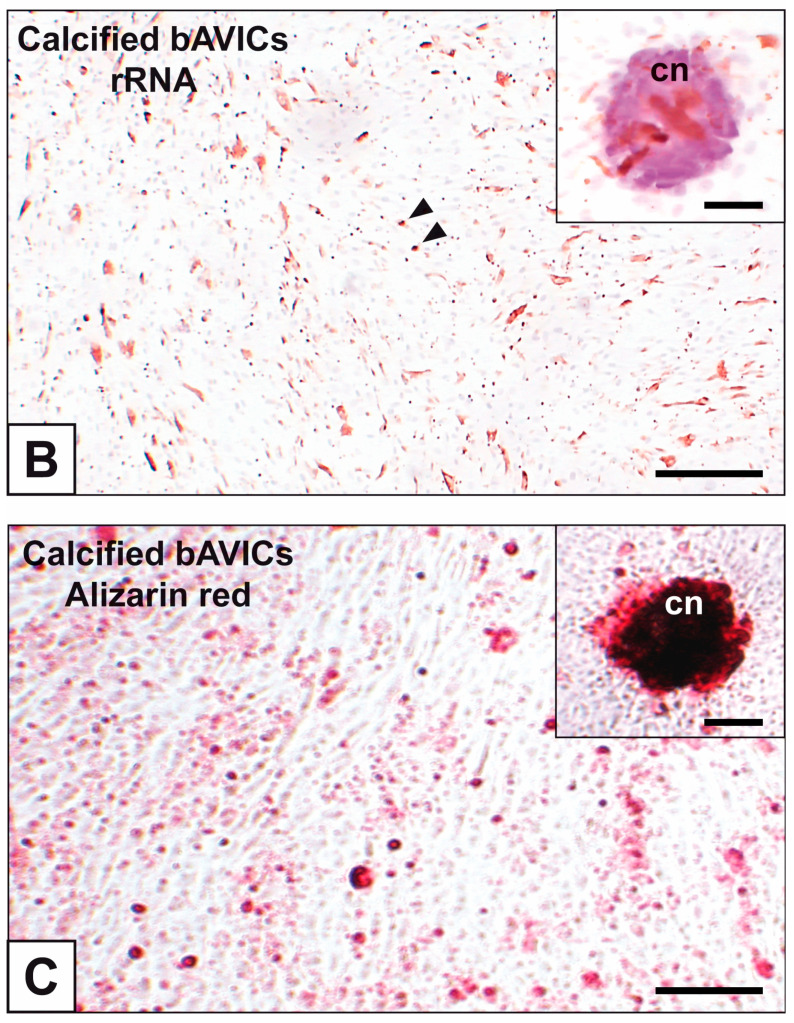
Immunocytochemical detection of rRNA in 15-day-long bovine aortic valve interstitial cell (bAVIC) cultures. (**A**) Immunopositivity of untreated bAVICs. (**B**) Immunopositivity of mineralizing bAVICs as well as vesicular cell debris (arrowheads) and calcific nodule areas (cn; inset). (**C**) Alizarin red calcium staining of mineralizing bAVICs and calcific nodules (cn; inset). Bar: 1 mm.

**Figure 2:**
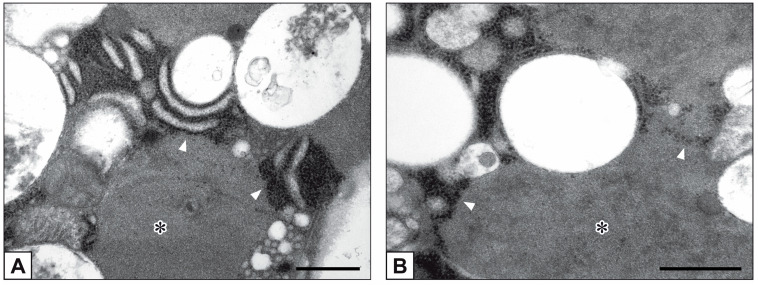

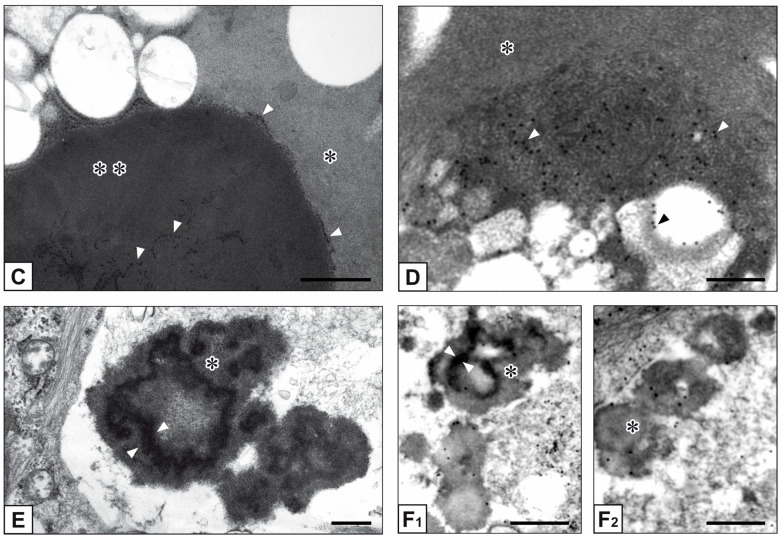
Ultrastructure of bAVICs from 15-day-long pro-calcific cultures. (**A**–**C**) Enlargement of RER *cisternae* (arrowheads) and RER membrane lysis, with ribosome release and merging with the nearby phthalocyanine-positive material (single asterisk: less condensed PPM; double asterisk: more condensed PPM) derived from ongoing membranous organelle degeneration. (**D**) Immunogold labelling of rRNA with gold particles (white arrowheads) decorating the intracellular PPM (asterisk) and a cross-sectioned degenerating RER *cisterna* (black arrowhead). (**E**) Cell-derived byproducts showing a mix of PPM (asterisk) and more electrondense phthalocyanine-positive layers (PPLs; counterposed arrowheads). (**F_1_**,**F_2_**) Immunogold labelling of rRNA showing gold particles within PPM (asterisks) and PPLs (counterposed arrowheads) at the level of vesicular cell debris. Bar: 0.5 μm (**A**–**C**,**E**); 0.25 μm (**D**,**F_1_**,**F_2_**).

**Figure 3:**
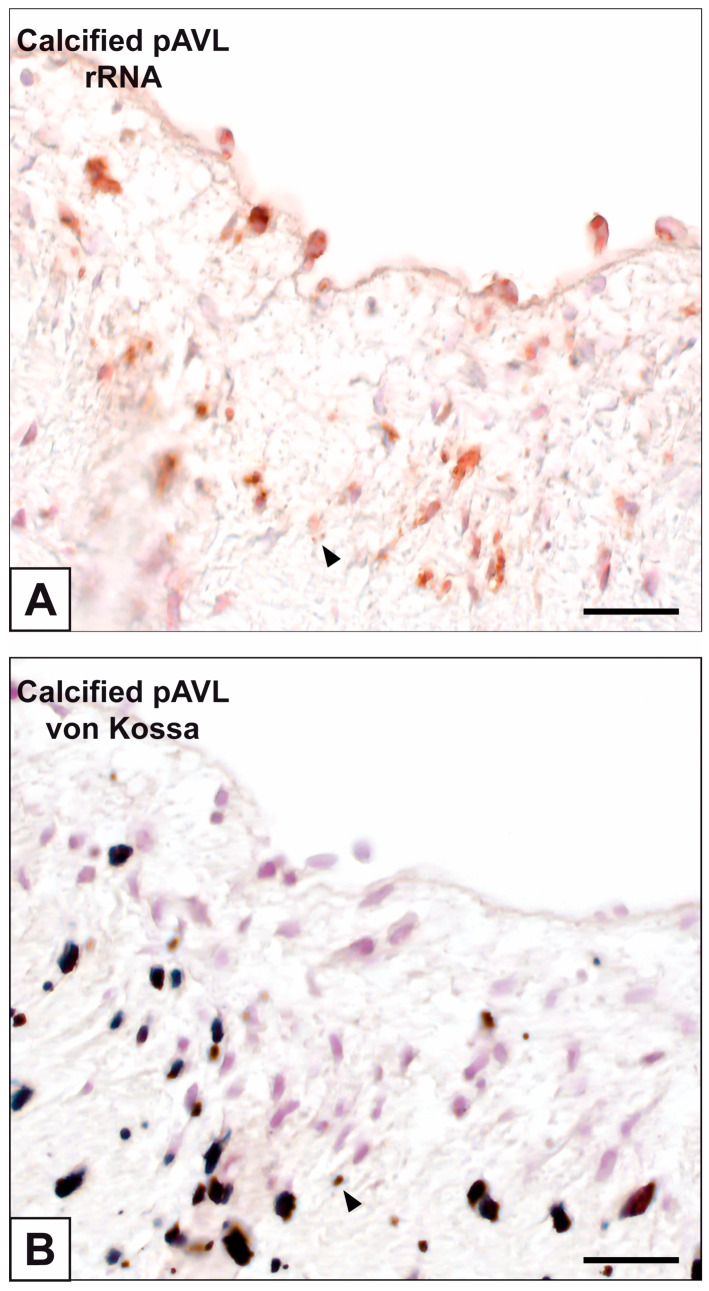
Immunohistochemical detection of rRNA in porcine aortic valve leaflets (pAVLs) after 7-day-long implantation in rat *subcutis*. (**A**) Immunopositivity of residual endothelial cells and mineralizing pAVICs as well as cell debris (arrowhead) in the *tunica fibrosa*. (**B**) Paraserial section showing positivity to von Kossa staining of mineralizing pAVICs and cell debris (arrowhead). Bar: 0.25 mm.

**Figure 4:**
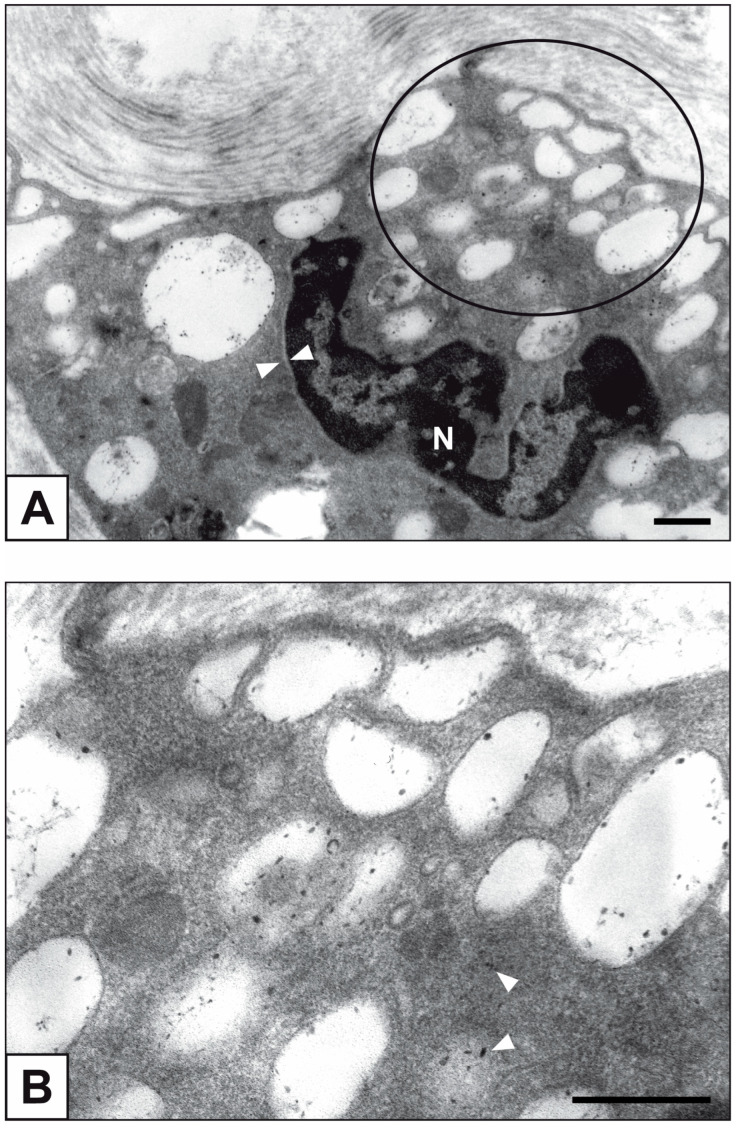
RER ultrastructure in AVICs populating 7-day-long-implanted pAVLs. (**A**) Enlarged RER *cisternae* (circle) in a mineralizing cell showing electrondense PPM and disappearance of nuclear envelope (counterposed arrowheads; N: nucleus). (**B**) Magnification of the cytoplasm area encircled in (**A**) showing release of ribosomes from dissolving RER membranes and melting with the nearby PPM (arrowheads). Bar: 0.5 μm.

**Figure 5:**
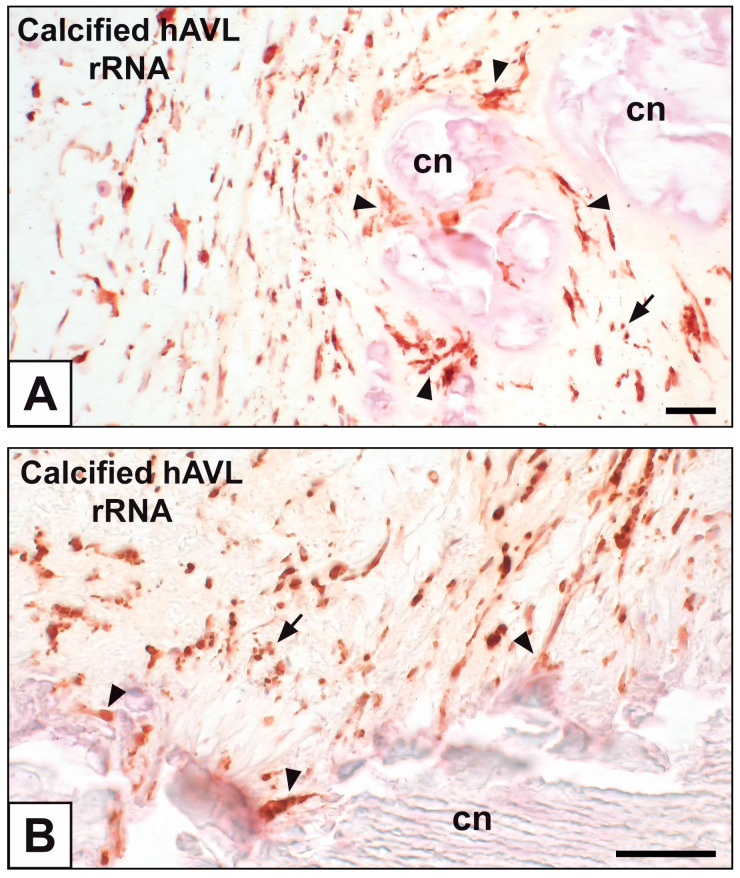
Immunohistochemical detection of rRNA in human stenotic aortic valve leaflets (hAVLs). (**A**,**B**) Immunopositivity of hAVICs, including those bordering calcific nodules (cn; arrowheads), and cell debris (arrows). Bar: 0.25 mm.

**Figure 6:**
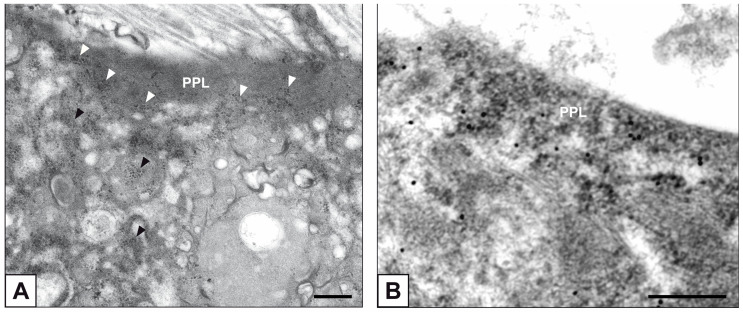

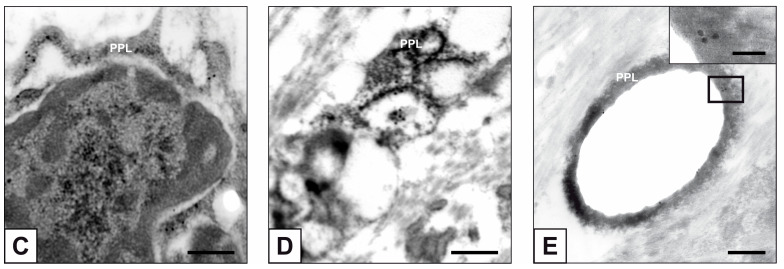
Ultrastructure of AVICs populating stenotic hAVLs. (**A**) Ribosomes (black/white arrowheads) embedded within PPM/PPL in a mineralizing hAVIC. (**B**,**C**) Immunogold labelling of rRNA with gold particles decorating peripheral PPLs. (**D**) Immunogold labelling of rRNA showing gold particles within PPM/PPLs in a hAVIC undergone advanced calcific degeneration. (**E**) Immunogold labelling of rRNA showing gold particles onto a PPL lining a vesicle-shaped cell byproduct. Inset: magnification of the PPL-squared region. Bar: 0.25 μm (**A**–**E**); 0.1 μm ((**E**) inset.

**Figure 7:**
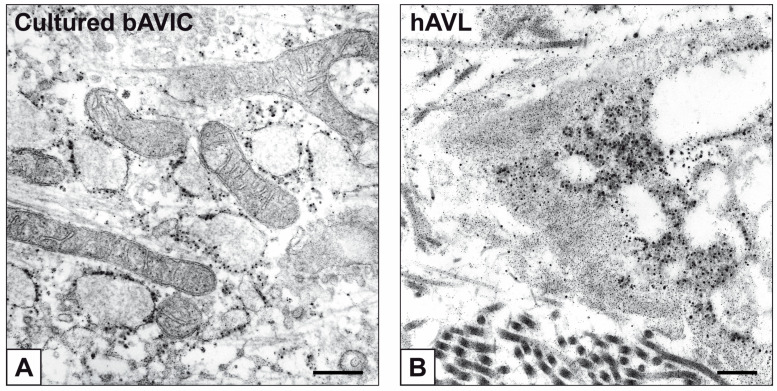

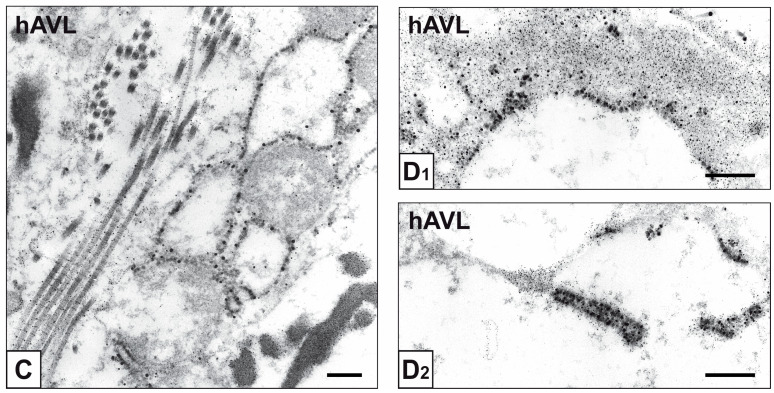
Post-embedding von Kossa silver staining of ribosomes in mineralizing AVICs. (**A**) Silver particle precipitation onto free ribosomes and those associated with RER membranes in a bAVIC at early calcification stages. (**B**–**D_2_**) Silver particle precipitation onto ribosomes associated with membranes lining enlarged RER *cisternae* or released nearby in hAVICs affected by ongoing calcific degeneration. Bar: 0.25 μm.

**Figure 8:**
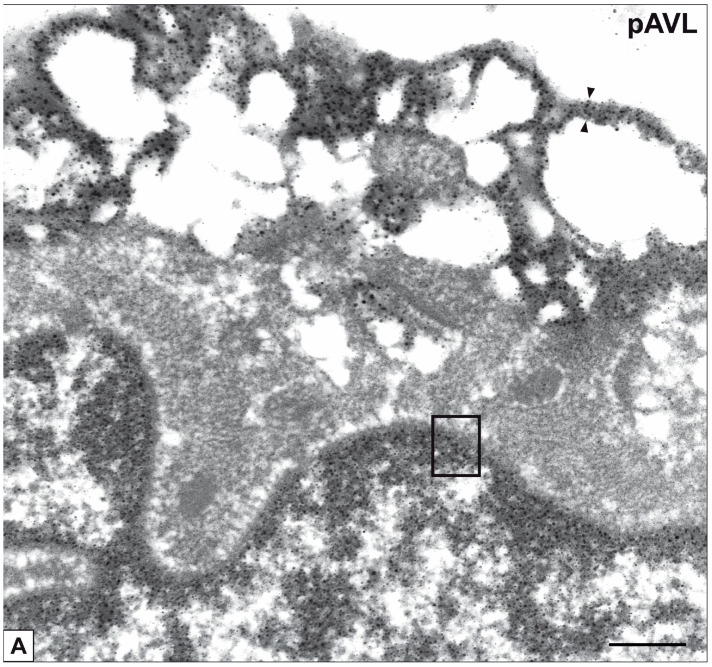

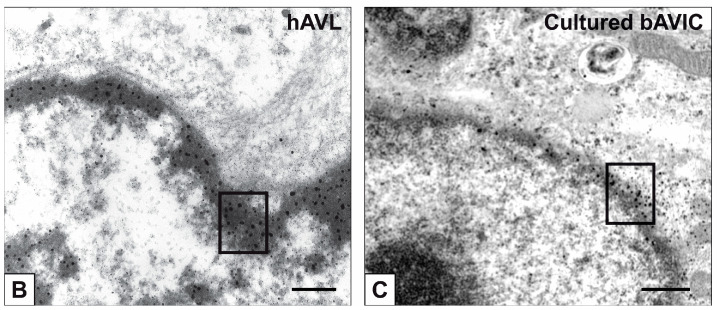
Post-embedding von Kossa staining of nuclear chromatin in mineralizing AVICs. Major silver particle precipitation onto nuclear heterochromatin (rectangular boxes) in mineralizing AVICs from (**A**) 28-day-long subdermally implanted pAVLs, with the largest silver particles being distributed along peripheral PPLs (counterposed arrowheads), (**B**) stenotic hAVLs, and (**C**) pro-calcific cultures. Bar: 0.25 μm.

Primary cultures of bAVICs were incubated for 9 to 28 days with or without pro-calcific *stimuli* to ascertain the potential involvement of nucleic acids in parallel with the calcification-stage progression. As revealed in light microscopy, lots of untreated bAVICs showed immunopositivity for rRNA over time (Figure 1A). Immunopositivity resulted also for several bAVICs cultured under pro-calcific conditions (Figure 1B), showing a slight decrease starting from day 21 because of advancing cell degeneration. Additional reactivity was exhibited by punctate vesicular cell debris as well as increasing numbers of calcific nodules (Figure 1B, 1B inset), which were stained by alizarin red and included mineralizing bAVICs, in parallel with pro-calcific cultures (Figure 1C, 1C inset). Ultrastructurally, mineralizing bAVICs showed a widespread disappearance of their endomembranes, including those lining the enlarged rough endoplasmic reticulum (RER) cisternae (Figure 2A–C) and nuclear envelopes. Many ribosomes released from the dissolving RER membranes were found to melt with the nearby electrondense PPM derived from overall membranous organelle degeneration (Figure 2A,B). In places, released or free ribosomes bordered and/or were embedded within a strongly electrondense PPM (Figure 2C), also appearing to be markedly decorated by gold particles after the immunogold labelling of rRNA (Figure 2D). PPM and even more electrondense PPLs lining degenerating bAVICs or their vesicular byproducts (Figure 2E) also exhibited superimposing particles after the immunogold labelling of rRNA (Figure 2F_1_,F_2_). Concerning pAVLs, many valve cells were immunopositive for rRNA in both native samples and those subjected to 2-day-long implantation (not shown). A slight decrease in immunopositive cells was appreciable after 7 implantation days (Figure 3A) because of the occurrence of mineralization. In more detail, immunopositivity involved residual endothelial cells as well as several mineralizing pAVICs and cell debris, consistently with their positivity to von Kossa silver staining on adjacent sections (Figure 3B). A remarkable decrease in immunopositive cells was apparent after 14 and 28 implantation days in association with massive tissue mineralization (not shown). As observed in vitro, ultrastructural analysis showed mineralizing pAVICs populating 2- and, to a greater extent, 7-day-long implanted pAVLs to exhibit the disappearance of nuclear envelopes and all other membranous components, including those lining enlarged RER *cisternae* (Figure 4A,B), with released ribosomes melting with PPM (Figure 4B). Consistently with more advanced cell degeneration, the intracellular content of pAVICs from pAVLs implanted for 14 and 28 days was entirely turned into a homogeneously electrondense PPM/PPL material, showing no more recognizable organelles (not shown). Immunopositivity for rRNA also involved several valve cells in both non-calcified and stenotic hAVLs. In the latter, immunopositivity was typically observed for mineralizing hAVICs and their vesicular debris bordering calcific nodules (Figure 5A,B). Additional immunopositivity involved residual endothelial cells and hAVICs populating leaflet areas that had still not undergone calcification. Ultrastructurally, mineralizing hAVICs also showed no or disappearing cell membranes, including those lining enlarged RER *cisternae*, with the releasing of associated ribosomes into the nearby PPM/PPLs (Figure 6A), as well as parallel decoration of the latter by gold particles after the immunogold labelling of rRNA (Figure 6B,C). Analogous immunopositivity was observed for both disaggregating AVICs (Figure 6D) and their vesicular debris (Figure 6E, 6E inset). In contrast with controls (not shown), after specific von Kossa reactions originally adapted for electron microscopy, ribosomes resulted as selective sites for silver particle precipitation irrespective of the calcific condition (Figure 7A–D_2_). Heterogeneously sized metal precipitates were observed to superimpose on ribosomes still associated with degenerating RER membranes as well as free ribosomes, sometimes forming spiral structures resembling polysomes. In addition to the expected positivity of PPM/PPLs for von Kossa reactions, silver particle deposition onto NC was also a common feature shared by mineralizing AVICs from all three calcific conditions (Figure 8A–C). Fine silver microprecipitates were detectable early at nuclear level in AVICs subjected to calcification induction both in vitro and in vivo. At more advanced calcification stages, the size and number of silver particles were clearly greater, mainly at the level of electrondense heterochromatin, although appearing slightly lower than those of particles precipitated onto PPM/PPLs. NC melting with PPM/PPL was found to occur at the latest calcification stages, showing no degenerative features characterizing apoptotic (chromatin margination/condensation/fragmentation) or oncotic (chromatin flocculation/pyknosis) cell death.

## 3. Discussion

In the present study, rRNA and NC were found to be involved in AVIC mineralization in three distinct conditions, i.e., (i) pro-calcific cultures, (ii) pAVLs subjected to calcific induction after implantation into rat *subcutis*, and (iii) hAVLs affected by calcific stenosis. Indeed, ultrastructural examination revealed that in mineralizing AVICs, ribosomes are released from dissolving RER membranes, becoming part of PPM/PPL pro-calcific substrates at the level of cells and cell-derived vesicular byproducts. It is of interest that Anderson had already reported the presence of ribosomes within vesicular debris released from mineralizing chondrocytes of mouse epiphyseal cartilage in 1969 [43], although their potential role in calcification was not addressed. The presence of ribosomes within PPM/PPLs was consistent with the superimposition on such pro-calcific material of gold particles after immunogold labelling of rRNA, besides the immunopositivity of mineralizing AVICs and their debris in light microscopy. The further superimposition of silver particles after specific von Kossa reactions indicates that ribosomes actually contribute to HA nucleation together with PPM/PPL substrates. Within the latter, the identification of ribosomes and the immunodetection of the inherent rRNA suggest that their degradation occurs much later than that of cell membranous components during AVIC calcification. Such an assumption is consistent with early cell membrane lysis due to an equally early expression of calcium-dependent cytosolic phospholipase A2α by mineralizing AVICs, as reported for both in vivo [35] and in vitro [38] experimental calcification. Since enzyme expression increased with time, the progressive turning of membranous cell components into homogeneously electrondense PPM/PPLs could reasonably explain the decreased numbers of rRNA-immunopositive AVICs in both pro-calcific cultures and pAVLs after the longest incubation times or subdermal confinement, respectively. Besides rRNA, NC was also found to contribute to HA nucleation during AVIC calcification, as indicated by the precipitation of metallic silver particles onto cell nuclei after post-embedding von Kossa reactions. As the size and number of silver particles precipitated onto the euchromatin were clearly smaller than those of particles precipitated onto the heterochromatin, a major contribution of the latter in HA nucleation is suggested. This is consistent with the fact that heterochromatin is more densely packed, exposing a higher number of negative charges per volume unit which are only in part neutralized by histone proteins [44]. Moreover, NC association with non-histone regulatory proteins rich in acidic amino acid residues [45] might explain for the identification of calcium-binding sites at a nuclear level. Interestingly, NC did not show typical features as described for apoptotic or oncotic cell death with calcification advancing, strengthening the concept that AVIC mineralization depends on the already described type of pro-calcific cell death characterized by overall cell membrane lysis with formation of acidic-lipid-containing pro-calcific substrates [35,37,38]. Concerning the size and number of the silver particles precipitated onto the NC, it is sufficient to point out that they were usually smaller than those precipitated onto PPM/PPLs, supporting the concept that major HA nucleation occurs at the level of such acidic-phospholipid-containing substrates. A pro-calcific role of nucleic acids in valve mineralization is also supported by evidence that the decellularization of native valves using a combination of their enzymatic removal with endonucleases and detergent-based membrane solubilization is recommended to attain durable, non-calcifiable valve biosubstitutes as a better alternative to glutaraldehyde-treated xenografts or cryopreserved allografts. Indeed, the additional use of endonucleases resulted in drastically reduced calcification in decellularized valve scaffolds [24,46,47], besides making them more permissive to cell repopulation [48,49,50,51]. In conclusion, the present study provides ultrastructural evidence that rRNA derived from ribosome degradation is also immunohistochemically detectable in the acidic-phospholipid-containing PPM/PPL pro-calcific substrates, contributing to HA nucleation during both experimental and pathological AVIC calcification. Nuclear chromatin acted as an additional HA nucleational site in all the examined calcific conditions, strengthening the relevance of nucleic acid removal to prevent calcification in valve scaffolds for aortic valve transplantation.

## 4. Materials and Methods

### 4.1. bAVIC Treatment

Primary cultures of bAVICs were obtained as previously described [33,36,37,38]. Bovine hearts were retrieved at slaughtering in a local abattoir (Salumificio Pitaccolo, Castions di Strada, Udine, Italy) respecting the protection of animals at the time of killing (Reg. CE 1099/2009, 24 September 2009). Cows were not killed specifically for the purpose of the present study and no experiments were performed on them before slaughtering. bAVICs were seeded onto 24 × 24 mm cover glasses placed into 35 × 10 mm Petri dishes and cultured for 9, 15, 21, and 28 days either under normal conditions (control) in Dulbecco’s Modified Eagle’s Medium (DMEM; Sigma-Aldrich, St. Louis, MO, USA) plus 10% fetal bovine serum (FBS; Gibco, Waltham, MA, USA) or pro-calcific conditions in DMEM (Sigma-Aldrich, St. Louis, MO, USA), plus 10% FBS (Gibco, Waltham, MA, USA), plus 2.0 mM inorganic phosphate, plus 100 ng/mL lipopolysaccharide from *E. coli* (LPS; Sigma-Aldrich, St. Louis, MO, USA), plus 1/5 (*v*/*v*) of conditioned medium obtained from LPS-stimulated bovine macrophages [33,36,37,38]. For conditioned medium preparation, bovine blood was collected by a veterinarian during routine care with respect for normal animal behavior and wellness, according to the professional ethics of FNOVI (Federazione Nazionale Ordini Veterinari Italiani) approved on 12 June 2011. Both culturing and stimulating media were refreshed every 3 days. All cultures were prepared in triplicate (*n* = 3).

### 4.2. Subdermally Implanted pAVLs

pAVLs were excised from aortic valves of pig hearts retrieved at slaughtering and then subjected to accelerated calcific induction by xenogeneic subdermal implantation in rats according to Schoen and colleagues [12] for 2, 7, 14, and 28 days (*n* = 3 for each time). Briefly, pAVLs were mildly fixed with 0.625% (*w*/*v*) glutaraldehyde prior subdermal implantation in the interscapular region of 3-week-old male Sprague–Dawley rats. Native pAVLs (*n* = 3) were used as control. No animals were killed specifically for the purpose of the present study, since stocks of embedded valve tissue samples were saved in the course of our previous investigations [29,30,31,32].

### 4.3. Stenotic hAVLs

hAVLs were excised from native tricuspid aortic valves surgically explanted from patients (*n* = 4; mean age = 78 ± 8 years) subjected to cardiac valve replacement at the Cardiothoracic Surgery Unit of the University Hospital of Udine. Severe, non-rheumatic calcific stenosis was diagnosed by preoperative clinical and echocardiographic parameters (valve area < 1 cm^2^; middle transvalvular gradient > 65 mmHg). Non-calcified aortic valve leaflets of hearts explanted from patients undergoing heart transplantation were used as control (*n* = 2). Analyses were performed in accordance with the ethical principles reported in the Declaration of Helsinki approved on June 1964 and its following revisions. Aortic valves for which patients previously signed an informed consent allowing explant use for scientific purposes were exclusively employed. The present study was approved by the Internal Review Board of the Department of Medicine of the University of Udine (Prot. 178/2022 approved on 7 December 2022).

### 4.4. Immunocytochemical Assay of rRNA in bAVIC Cultures

Cell cultures were fixed with 3% phosphate-buffered paraformaldehyde for 10 min and then treated with: (i) 0.1% Triton X-100 for 10 min; (ii) 3% hydrogen peroxide for 5 min; (iii) 3% normal serum for 40 min; (iv) 1:200 mouse anti-rRNA primary antibody (Santa Cruz Biotechnology, Dallas, TX, USA) for 90 min at room temperature; (v) 1:100 peroxidase-conjugated anti-mouse antibody (Santa Cruz Biotechnology, Dallas, TX, USA) for 30 min; and (vi) DAB chromogen (BioGenex, Fremont, CA, USA) prepared according to the manufacturer’s instructions for 6 min. As control, the primary antibody was replaced with normal serum. After mild counterstaining with hematoxylin, cover glasses were mounted onto microscope slides using an aqueous mounting medium. Photographic recording was made using an AxioImager photomicroscope (Carl Zeiss, Oberkochen, Germany).

### 4.5. Alizarin Red S Calcium Staining of bAVIC Cultures

Cell cultures were fixed with 5% phosphate-buffered formalin for 10 min and then treated with an aqueous solution of 2% alizarin red S (Carlo Erba Reagents, Milano, Italy), pH 4.2, for 5 min. After rinsing with distilled water, cover glasses were mounted onto microscope slides using an aqueous mounting medium. As a control, parallel pro-calcific bAVIC cultures were subjected to decalcification before staining by incubation in a 0.05 M sodium acetate/acetic acid buffer, pH 4.8, for 1 h at room temperature. Photographic recording was undertaken using the AxioImager photomicroscope as above.

### 4.6. Immunohistochemical Detection of rRNA in AVLs

Histological sections obtained from paraformaldehyde-fixed, paraffin-embedded pAVLs and hAVLs were deparaffinised, rehydrated, and subjected to steps (i)–(vi) as described above, for immunocytochemical analysis of cultured bAVICs. After mild counterstaining with hematoxylin, histological sections were dehydrated, soaked in xylene, and mounted with Eukitt^®^ mounting medium. Photographic recording was performed using the Zeiss AxioImager photomicroscope as above.

### 4.7. Von Kossa Silver Staining for Calcium Binding Site Visualization in AVLs

Histological sections of pAVL and hAVL samples were deparaffinised, re-hydrated, and incubated with 1% silver nitrate for 15 min under direct sunlight. After rinsing with distilled water, sections were incubated with 5% sodium thiosulphate for 5 min, rinsed again, and mildly counterstained with hematoxylin. Following this, sections were de-hydrated, soaked in xylene, and mounted with Eukitt^®^ mounting medium. Photographic recording was performed using the Zeiss AxioImager photomicroscope as above.

### 4.8. Transmission Electron Microscopy

Cell cultures, as well as pAVL and hAVL samples, were fixed with 2.5% glutaraldehyde diluted in 25 mM sodium acetate/acetic acid buffer, pH 4.8, containing 0.05% phthalocyanine cuprolinic blue (Electron Microscopy Sciences, Hatfield, PA, USA) and 0.05 M magnesium chloride, overnight at room temperature under constant stirring. Following this, bAVIC cultures and AVL samples were: (i) post-fixed with 2% osmium tetraoxide (Agar Scientific, Stansted, Essex, UK), (ii) dehydrated with graded ethanol solutions, and (iii) embedded into Epon 812 resin or Epon-Araldite resin, respectively. Ultrathin sections were collected onto formvar-coated 2 × 1-mm-slot copper grids and contrasted with uranyl acetate and lead citrate. Observations and photographic recording were undertaken using a CM12 STEM transmission electron microscope (Philips, Eindhoven, The Netherlands).

### 4.9. Immunogold Labelling of rRNA

Cell cultures, as well as pAVL and hAVL samples, were fixed with phosphate-buffered 4% paraformaldehyde, dehydrated with graded ethanol solutions, and embedded into LR-white resin. Following this, ultrathin sections were incubated with: (i) 5% normal serum for 30 min; (ii) 1:200 mouse anti-rRNA primary antibody (Santa Cruz Biotechnology, Dallas, TX, USA) overnight at +4 °C; and (iii) 1:15 anti-mouse gold-conjugated secondary antibody (Jackson Immunoresearch, Ely, UK) for 60 min at room temperature. As control, primary antibody was replaced with normal serum. After weak contrasting with uranyl acetate and lead citrate, observations and image recording were undertaken using the Philips CM12 STEM electron microscope as above.

### 4.10. Post-Embedding von Kossa Silver Staining for Ultrastructural Calcium Binding Site Visualization

Semithin sections of Epon-embedded bAVIC cultures as well as pAVL and hAVL samples were incubated with 1% silver nitrate for 15 min under direct sunlight, keeping glass slides on a plate warmed at +80 °C. After rinsing with distilled water, semithin sections were incubated, warm, with 5% sodium thiosulfate for 5 min. Top-less conic BEEM capsules were then glued onto the glass slides, so encircling each reacted semithin section, and filled with fluid Epon resin for section re-embedding. Ultrathin sections were collected onto formvar-coated 2 × 1-mm-slot copper grids and weakly contrasted with uranyl acetate and lead citrate. Observations and image recording were made using the Philips CM12 STEM electron microscope as above.

## Data Availability

Data is contained within the present article. Supplementary images not shown in this study are available on request to the corresponding authors.
